# Acute lorazepam administration does not significantly affect moral attitudes or judgments

**DOI:** 10.1038/s41598-025-01109-9

**Published:** 2025-05-08

**Authors:** Róger Marcelo Martinez, Li Wei, Yang-Teng Fan, Yu-Chun Chen, Kah Kheng Goh, Yen-Nung Lin, Cheng-Ta Yang, Yawei Cheng, Chenyi Chen

**Affiliations:** 1 Graduate Institute of Injury Prevention and Control, College of Public Health, Taipei Medical University, Taipei, Taiwan; 2 Division of Neurosurgery, Department of Surgery, Wan Fang Hospital, Taipei Medical University, Taipei, Taiwan; 3 School of Psychological Sciences, National Autonomous University of Honduras, Tegucigalpa, Honduras; 4 Research Center for Neuroscience, Taipei Medical University, Taipei, Taiwan; 5 Graduate Institute of Medicine, Yuan Ze University, Taoyuan, Taiwan; 6 Department of Physical Education, National Taiwan University of Sport, Taichung, Taiwan; 7 Psychiatric Research Center, Wan Fang Hospital, Taipei Medical University, Taipei, Taiwan; 8 The Innovative and Translational Research Center for Brain Consciousness, Taipei Medical University, Taipei, Taiwan; 9 Department of Psychiatry, Wan-Fang Hospital, Taipei Medical University, Taipei, Taiwan; 10 Department of Physical Medicine and Rehabilitation, Wan Fang Hospital, Taipei Medical University, Taipei, Taiwan; 11 Department of Psychology, National Cheng Kung University, Tainan City, Taiwan; 12 Department of Physical Medicine and Rehabilitation, National Yang Ming Chiao Tung University Hospital, Yilan, Taiwan; 13 Institute of Neuroscience, National Yang Ming Chiao Tung University, Taipei, Taiwan; 14 Graduate Institute of Injury Prevention and Control, Taipei Medical University, 250 Wu-Hsing Street, Taipei City, 110 Taiwan; 15 Institute of Neuroscience, National Yang Ming Chiao Tung University, No. 155, Sec. 2, St. Linong, Dist. Beitou 112, Taipei , Taiwan, ROC; 16 Department of Education and Humanities in Medicine, College of Medicine, Taipei Medical University, Taipei, Taiwan

**Keywords:** Lorazepam, Moral judgement, Implicit moral attitudes, Justice sensitivity, Gender differences, Neurophysiology, Cognitive neuroscience, Emotion, Social behaviour, Social neuroscience

## Abstract

Recent scientific studies exploring the neuropsychological foundations of moral decision-making have shown that moral attitudes and evaluations are significantly influenced by emotion, particularly negative emotionality, as well as personality traits such as neuroticism. Further psychopharmacological research has observed that GABAergic agonists are capable of influencing moral decision-making by modifying anxiety-related emotional negativity and/or through cognitive modulation. The aim of this double-blind, crossover design, placebo-controlled study is to evaluate said GABAergic modulation on moral cognition. Importantly, unlike the aforementioned research, the present study not only utilizes explicit moral evaluation measures [e.g., the Justice Sensitivity Inventory (JSI) and evaluations of moral scenarios], but also uses the morality Implicit Association Test (mIAT) to assess unacknowledged attitudes towards morally charged scenarios. Our results indicate that acute lorazepam administration does not significantly alter moral judgments, including implicit moral attitudes, explicit justice sensitivity, and blame/praise evaluations. Lorazepam-induced changes in moral sensitivity appeared to vary with baseline levels, with individuals exhibiting higher baseline JSI or D scores tending to show greater reductions following administration. These findings support the involvement of GABAergic modulation in moral cognition, albeit without clear behavioral consequences.

## Introduction

Recent scientific literature shows that emotions, particularly negative ones like anxiety—as well as neuroticism, a personality trait—significantly influence moral decision-making^1^. These findings reveal that moral attitudes are deeply intertwined with affective responses alongside logical deliberation^2,3^.

Negative emotionality and neuroticism affect a person’s likelihood to avoid antisocial behaviors^4^. Individuals who are prone to risk aversion and are strongly influenced by social pressures tend to have higher levels of neuroticism^5,6^. This is because the anxiety and negative affect they experience make them more likely to avoid uncertain situations^4^. Furthermore, conflicts with others—a known trigger for anxiety—further amplify this tendency^7,8^. The instinctive “gut” reaction many people feel when considering harming another person, even if it might serve a greater good, is now understood as a physical manifestation of emotional negativity and anxiety^9^.

Moral decision-making is believed to be influenced by mechanisms similar to those used in risk-based decision-making^10^, as these decisions are often made without knowing their outcomes in advance^11^. Nevertheless, while the outcomes of deontological judgments, which are more rule-based, are easier to be inferred, those associated with utilitarian choices—decisions that prioritize the greater good independently of the means to get to it—are not, as the psychological burden (e.g., potential regret and guilt) cannot be predicted. This renders utilitarian judgments more akin to decisions made under risk and uncertainty, similar to gambling^10,11^.

Moreover, research suggests that strategies to regulate emotions or reduce emotional reactivity can lead to a preference for risky and utilitarian decisions by decreasing negative affect^3,12^. Conversely, increasing negative emotionality, linked to serotonergic activity in the brain, tends to inhibit utilitarian choices^1^. Theoretical models of decision-making under uncertainty propose that people are more likely to choose risky or utilitarian options when the potential rewards outweigh the anxiety associated with uncertainty, or when they have the cognitive capacity to suppress moral and emotional responses. Enhanced cognitive ability allows for more rational navigation through moral decision-making^13,14^.

Clinical observations suggest that anxiety significantly influences the likelihood of choosing harmful actions in moral dilemmas. A study, demonstrated how even inducing anxiety in a mild manner, prompted participants to rate morally worse the utilitarian option in both the switch and the footbridge version of the trolley moral dilemma, even in participants that had previously rated the switch version as morally good^10^. However, it is important to note that these findings are based on correlational data, which do not establish a direct causal link between anxiety and moral decision-making^3^.

Furthermore, it has been observed that several neurotransmitters and neuropeptides incur in moral decision-making by way of emotional modulation. Two of said neuropeptides are oxytocin and vasopressin, both of which have been implicated in emotional and social responses: anxiety and stress coping in the former; and aggression, empathy, and parental care (among others) in the latter. Moreover, it has been observed that oxytocin and vasopressin modulate and are modulated by the dopaminergic system, such that their influence is bidirectional^15^. Regarding the role of the dopaminergic system in moral decision-making, some studies show that increased dopamine levels reduce hyperaltruism—the tendency to prioritize others’ suffering over one’s own^16^; while others found no link between dopaminergic activity and deontological versus utilitarian judgements^17^. In Parkinson’s disease patients, dopaminergic hypoactivity has been linked to patients within the hyper-honest subgroup (who demonstrate less propensity towards deceptive tendencies than neurotypical controls), while dopaminergic hyperactivity was associated with those in the hypo-honest subgroup (often characterized by comorbidities with impulse control disorders). These patterns appear to reflect dysfunctions in the dopaminergic reward system, influencing the motivation to deceive^18^. Additionally, research indicates that serotonin upregulation enhances moral judgment by increasing harm aversion^19^. Taken together, oxytocin, vasopressin, the serotonergic and dopaminergic systems jointly modulate moral cognition and emotional responses to moral stimuli, influencing moral decision-making through distinct effects^15,16^.

Lorazepam, a high-potency benzodiazepine, is commonly prescribed to relieve anxiety symptoms^20^. It enhances GABA activity in the brain, a neurotransmitter that inhibits neural activity, through binding to GABA receptors. Research shows that lorazepam increases participants’ willingness to endorse harmful actions in moral dilemmas, whether for selfish or utilitarian reasons^3^. This suggests that anxiolytic drugs like lorazepam may affect moral decision-making by reducing the emotional negativity associated with anxiety and modulating cognitive processes^12,21^.

This double-blind, crossover design, placebo-controlled study aims to evaluate how lorazepam influences moral evaluation and attitudes, addressing limitations in previous research. Earlier studies^3^ relied on questionnaires and other self-reported measures probing explicitly for moral attitudes. Nevertheless, this approach leaves the accuracy of these instruments subject to social desirability biases^22^; thus, susceptible to the participants’ ability to intentionally alter or mask their genuine responses.

In the current research, explicit measures of moral attitudes, such as the Justice Sensitivity Inventory (JSI), are used. The JSI distinguishes between self-oriented and other-oriented (utilitarian) justice sensitivities and moral attitudes. Additionally, the study incorporates the morality version of the Implicit Association Test (mIAT) to assess individuals’ involuntary attitudes toward morally charged scenarios^23,24^. The mIAT measures the speed and accuracy with which participants categorize concepts, like associating “moral” versus “immoral” actions with evaluative attributes. Participants are presented with congruent pairs (e.g., moral scenarios paired with positive words, and immoral scenarios paired with negative words) and incongruent pairs (e.g., moral scenarios paired with negative words, and immoral scenarios paired with positive words). Differences in reaction times between these conditions arise because congruent pairs align with automatic cognitive associations, leading to faster responses, while incongruent pairs demand greater cognitive effort, resulting in slower reaction times. Faster and more accurate associations suggest a stronger link between these concepts in memory, while slower associations suggest a weaker connection. By analyzing these reaction time differences, the mIAT provides insight into the automatic cognitive processes underlying moral evaluations, offering a valuable complement to explicit measures, which are often subject to social desirability biases and deliberate self-presentation concerns. This approach allows researchers to explore the subtle and unconscious aspects of moral cognition that inform ethical behavior^23^.

Our previous studies have demonstrated that implicit moral attitudes, like other implicit attitudes, are inherently unstable and can be modulated by situationally induced changes. Even in the absence of experimental manipulations, the test-retest reliability of the Implicit Association Test (IAT) remains relatively low (*r* = 0.5–0.6) over time, particularly when compared to conceptually corresponding explicit measures (weighted average *r* = 0.75) in purely longitudinal assessments^25^. Similarly, test-retest reliability declined further when modulatory factors were introduced. This instability may be attributed to two key factors: (1) the inherently low test-retest reliability of the IAT and (2) the IAT’s heightened sensitivity to modulatory influences, with effects likely varying across individuals rather than being uniformly distributed. For instance, the modulation of the mIAT is highly dependent on individual characteristics, such as baseline attitudes, even in the absence of external interventions. Social factors—including the presence of others and variations in perceived agency (actor vs. observer)—have been shown to influence both implicit moral attitudes (△D in mIAT) and justice sensitivity (△JSI). These social modulation effects further vary as a function of participants’ baseline characteristics, such as initial D and JSI scores^26,27^. Considering the role of lorazepam-induced social modulation in moral decision-making and associated neural mechanisms^12,28^, we hypothesize that acute lorazepam administration will impact implicit moral attitudes and justice sensitivity based on baseline features, such as mIAT and JSI scores, under placebo conditions. This aligns with previous studies^26,27^, which highlight the interactions between baseline characteristics and social factors, leading to varying levels of influence.

Specifically, building on previous findings demonstrating that lorazepam reduces inhibitions in moral dilemmas, thereby increasing the likelihood of utilitarian choices, this study hypothesizes that lorazepam will influence both explicit and implicit moral attitudes, with a more pronounced effect on implicit attitudes—driven by automatic emotional processes^3,12^. Particularly, lorazepam is expected to reduce negative emotionality potentially elicited by harm-related scenarios, which may, in turn, diminish the incongruent conflict between positive valence and morally transgressive actions. This effect would lead to reduced D scores (as reflected in changes to D scores rather than overall reaction times on the mIAT), lower other-oriented justice sensitivity, and altered subjective moral evaluations.

## Materials and methods

### Participants

Eighty-one healthy volunteers (40 men), aged between 21 and 31 years (mean ± SD: 23.556 ± 2.29), participated in the study after providing written informed consent. All participants were Han Chinese and right-handed. They underwent screening for major psychiatric illnesses (e.g., general anxiety disorder) using the Structured Clinical Interview for DSM-IV Axis I Disorders (SCID-I) and were excluded if they showed evidence of comorbid neurological disorders (e.g., dementia, seizures), had a history of head injury, or had alcohol or substance abuse or dependence within the past five years. All participants had normal or corrected-to-normal vision and were not taking any medication at the time of the study. None of the female participants were using oral contraceptives. This study was approved by the Ethics Committee of National Yang-Ming University (YM104041E) and conducted in accordance with the Declaration of Helsinki.

## General procedures

In a double-blind, placebo-controlled, crossover design, participants received a single 0.5-mg dose of lorazepam (ATIVAN) on one day, and a single dose of placebo (i.e., vitamin C) on another day. Participants underwent the same experimental procedure for both the placebo and lorazepam administrations, with at least a two-week washout period between sessions. Both lorazepam and placebo were administered orally. The sequence of lorazepam and placebo administration was counterbalanced between participants through a Latin square design, which ensures an equal number of AB (lorazepam-placebo) and BA (placebo-lorazepam) sequences. To align with the pharmacokinetics of lorazepam^29^, the behavioral assessment took place approximately 2 h after treatment administration. After drug or placebo administration, participants completed the Justice Sensitivity Inventory (JSI) and the subjective evaluation of morally laden scenarios task to assess their explicit moral attitudes, and performed the moral Implicit Association Task (mIAT) to measure their implicit moral attitudes.

## Implicit moral attitude (mIAT)

The mIAT was adapted with animations and words as stimuli. The verbal stimuli comprised 26 extremely pleasant and 26 extremely unpleasant words, selected from commonly used Chinese words^26,30,31^. The animated stimuli included 47 clips depicting everyday dyadic interactions, where each action was characterized as moral or immoral based on outcomes of personal assistance or harm, respectively^27,32^ (see Supplementary Fig. 1 for an example of the visual stimuli). The mIAT followed the experimental design outlined by Greenwald et al.^23^, consisting of five discrimination blocks:

- Block 1, ‘initial target-concept discrimination,’ has participants categorizing clips as moral (right response key) or immoral (left response key).
- Block 2, ‘attribute discrimination,’ involves categorizing words as negative (right response key) or positive (left response key).
- Block 3, ‘combined target-concept discrimination,’ presents clips and words in alternating trials, with moral clips paired with negative words (right response key) and immoral clips with positive words (left response key).
- Block 4, ‘reversed target-concept discrimination,’ reverses the response assignment from Block 1, with moral clips (left response key) and immoral clips (right response key).
- Block 5, ‘combined reversed target-concept discrimination,’ pairs immoral clips with negative words (right response key) and moral clips with positive words (left response key).

The sequences of the blocks (12345/42513) were counterbalanced to mitigate sequential effects. Half of the participants first encountered the incongruent block, and the other half started with the congruent block. Blocks 1, 2, and 4 consisted of 20 trials each, while Blocks 3 and 5 contained 40 trials each, with words and/or clips randomized within each block. Each trial began with a 1000-ms fixation cross, followed by a stimulus, presented on a computer using E-Prime version 2.0 software (Psychology Software Tools). Participants were instructed to categorize each word or clip as quickly and accurately as possible, with ten practice trials preceding Blocks 3 and 5. Accuracy rates and reaction times (RT) for Blocks 3 and 5 were recorded.

Performance on the mIAT is quantified using D scores, which are calculated by subtracting the mean reaction time (RT) of congruent (moral-negative) blocks from that of incongruent (moral-positive) blocks, and dividing the result by the pooled standard deviation of the two blocks^33^ As both the numerator and denominator are measured in milliseconds, the resulting D score is a dimensionless (unitless) value. Higher D scores indicate stronger implicit moral attitudes, reflected by longer RTs for incongruent blocks or shorter RTs for congruent blocks.

To account for the sensitivity of reaction time (RT) differentials to outliers and extreme values, RTs exceeding two standard deviations from the participant’s individual mean within each block were excluded as outliers, comprising less than 3% of all responses. This exclusion was performed separately for each discrimination block to address block-specific variability. Additionally, RTs faster than 300 ms or slower than 6000 ms, considered extreme and indicative of inattention, were excluded from analysis, accounting for less than 1% of all responses. The remaining RTs were used to compute differential scores, ensuring consistency and accuracy in the analysis^33^.

## Explicit measure of justice sensitivity: the justice sensitivity inventory (JSI)

The Justice Sensitivity Inventory (JSI) is a self-report psychometric tool designed to assess an individual’s sensitivity to unjust situations^34^. The inventory encompasses four distinct perspectives—beneficiary, observer, perpetrator, and victim—with each perspective comprising 10 items. Respondents rate these items on a six-point scale ranging from 0 (‘Not at all’) to 5 (‘Exactly’). The resultant scores reflect an individual’s sensitivity to moral norm violations and perceptions of injustice. Scores from the beneficiary, observer, and perpetrator perspectives are often aggregated to form a composite measure of other-oriented sensitivity^35^. This dimension of justice sensitivity is associated with traits such as high agreeableness, conscientiousness, and empathy. In contrast, a self-oriented justice sensitivity tends to correlate with higher levels of neuroticism and lower levels of agreeableness (see Supplementary Table 1 for an example of the questions).

## Subjective evaluations on morally laden scenarios

A set of 45 validated animations from previous fMRI studies, depicting ecologically valid dyadic social interactions, was presented^26,27,36^. Each animation comprised three images, set to durations of 1000 milliseconds for the first image, 200 milliseconds for the second, and 1000 milliseconds for the third, portraying scenarios of: [1] a person alleviating another’s physical pain (helping), [2] a person inflicting physical harm on another (harming), and [3] baseline stimuli depicting an action irrelevant to another person (neutral). The protagonists’ faces were obscured to prevent any emotional reactions from being visible to participants. The order of scenarios was randomized for each participant. In each animation trial, participants were asked to assess moral reasoning via a computer-based visual analogue scale. They rated each scenario on a Likert scale from “very much blameworthy (− 7)”, through “neutral (0)”, to “very much praiseworthy (7) [see Supplementary Fig. 2 for an example of the visual stimuli].

## Results

### Implicit moral attitude (mIAT) results

**Fig. 1:**
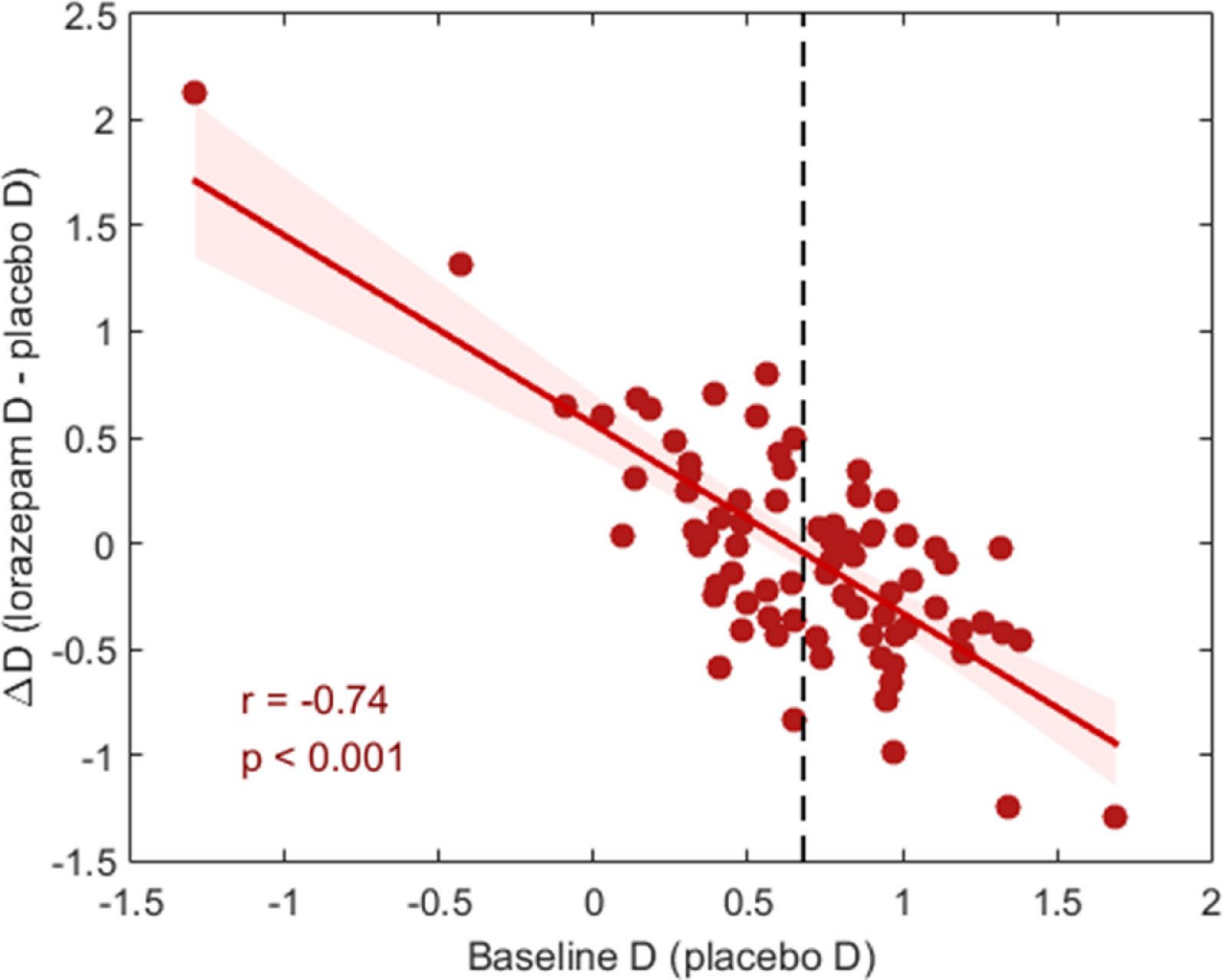
Lorazepam effects on implicit moral attitudes (mIAT) as a function of baseline D scores. Baseline D scores in the placebo condition were significantly correlated with △D (*r* = − 0.74, *p* < 0.001).

In this cohort, the D scores ranged from − 1.29 to 1.69, with mean and standard deviation of 0.68 ± 0.43 in the placebo condition, and from − 0.19 to 1.36, with mean and standard deviation of 0.63 ± 0.35 in the lorazepam condition (Table 1). While acute lorazepam administration did not significantly affect overall D scores [t(80) = 0.779, *p* = 0.438], we conducted a univariate ANCOVA to examine whether lorazepam-induced changes in implicit moral attitudes (△D, defined as lorazepam D minus placebo D) were associated with gender and baseline D. The model included △D as the dependent variable [F(3, 77) = 31.67, *p* < 0.001, partial eta squared (pη²) = 0.55], with gender as the fixed independent factor and baseline D (i.e., placebo D) and order of administration as covariates. The results revealed that baseline D significantly predicted △D [F(1, 77) = 94.8, *p* < 0.001, pη² = 0.55], whereas neither gender (*p* = 0.916) nor order of administration (*p* = 0.976) had a significant effect on △D (see Fig. 1). (Fig. 1).

**Table 1:** Descriptive statistics in placebo and lorazepam conditions. Notes: se = standard error; JSI: justice sensitivity inventory.

	Placebo	Lorazepam			
Measurements	Mean ± SE	Mean ± SE	T value	df	P value
JSI total score	17.2111 ± 0.3548	17.5346 ± 0.34088	-1.832	80	0.071
Self-oriented JSI	4.5074 ± 0.1054	4.5246 ± 0.1019	-0.272	80	0.787
Other-oriented JSI	4.2345 ± 0.0979	4.3367 ± 0.0947	-1.943	80	0.056
Implicit moral attitude (mIAT)	0.6789 ± 0.0476	0.6344 ± 0.0385	0.779	80	0.438
Blameworthy for harming	-5.0592 ± 0.1291	-5.1506 ± 0.1399	0.768	80	0.445
Praiseworthy for helping	4.4988 ± 0.1412	4.507 ± 0.1482	-0.078	80	0.938

## Justice sensitivity (JSI) results

**Fig. 2:**
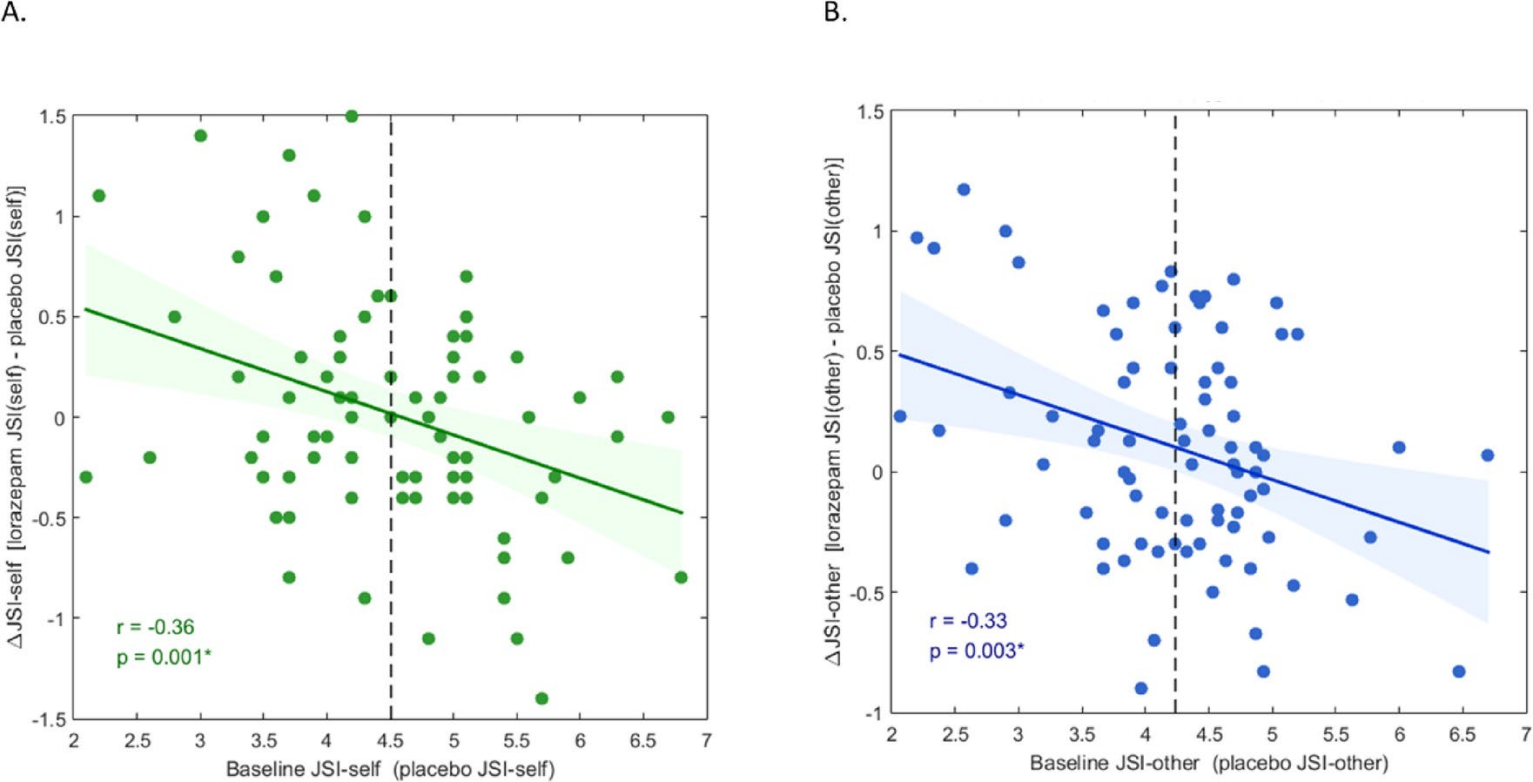
Lorazepam effects on justice sensitivity (JSI) as a function of baseline scores. (**A**) Baseline JSI-self scores in the placebo condition were significantly correlated with △JSI-self (*r* = − 0.36, *p* = 0.001). (**B**) Baseline JSI-other scores in the placebo condition were significantly correlated with △JSI-other (*r* = − 0.33, *p* = 0.003).

In this cohort, total JSI scores ranged from 8.8 to 26.4 with a mean and standard deviation of 17.21 ± 3.19 in the placebo condition, whereas in the lorazepam condition, scores ranged from 8.5 to 26.8 with a mean and standard deviation of 17.53 ± 3.07. For the self-oriented Justice Sensitivity Index (JSI), while acute lorazepam administration did not significantly affect overall self-oriented JSI scores [t(80) = -0.272, *p* = 0.787], a univariate ANCOVA was conducted to examine factors influencing lorazepam-induced changes. The model included △JSI-self (defined as lorazepam JSI-self minus placebo JSI-self) as the dependent variable [F(3, 77) = 3.869, *p* = 0.012, partial eta squared (pη²) = 0.131], with gender as the fixed independent factor, and baseline JSI-self (i.e., placebo JSI-self) and order of administration as covariates. Results indicated that baseline JSI-self significantly predicted △JSI-self [F(1, 77) = 10.376, *p* = 0.002, pη² = 0.119] (Fig. 2A), whereas neither gender (*p* = 0.604) nor order of administration (*p* = 0.768) had a significant effect on △JSI-self.

For the other-oriented Justice Sensitivity Index (JSI), acute lorazepam administration showed a trend toward reducing overall other-oriented JSI scores, although this effect did not reach statistical significance [t(80) = -1.943, *p* = 0.056]. To further investigate potential modulators of lorazepam-induced changes, a univariate ANCOVA was conducted with △JSI-other (defined as lorazepam JSI-other minus placebo JSI-other) as the dependent variable [F(3, 77) = 3.492, *p* = 0.020, partial eta squared (pη²) = 0.12]. Gender was included as the fixed independent factor, and baseline JSI-other (i.e., placebo JSI-other) and order of administration were included as covariates. The analysis revealed that baseline JSI-other significantly predicted △JSI-other [F(1, 77) = 8.78, *p* = 0.004, pη² = 0.102] (Fig. 2B), whereas neither gender (*p* = 0.363) nor order of administration (*p* = 0.754) had a significant effect on △JSI-other.

### Subjective moral evaluations

The 2 (DRUG: placebo vs. lorazepam; a within-subject factor) x 2 (GENDER: male vs. female; a between-subject factor) x 2 (SCENARIO: harming vs. neutral vs. helping; a within-subject factor) mixed ANOVA on subjective moral evaluations showed a main effect of scenario (F(2, 158) = 1324.759, *p* < 0.001, pη^2^) = 0.944; Harming vs. Neutral vs. Helping: -5.103 ± 0.12 vs. 1.208 ± 0.096 vs. 4.502 ± 0.135, mean ± standard error), while the main effects of drug (F(1, 79) = 0.656, *p* = 0.42, pη^2^ = 0.008) and gender (F(1, 79) = 2.951, *p* = 0.09, pη^2^ = 0.036) did not reach significance, nor did any interactions with drug or gender (all *p* > 0.2). The harming scenario was rated significantly more blameworthy than the neutral scenario (-5.103 ± 0.12 vs. 1.208 ± 0.096, *p* < 0.001), while the helping scenario was evaluated as significantly more praiseworthy compared to the neutral scenario (4.502 ± 0.135 vs. 1.208 ± 0.096, *p* < 0.001).

### Implicit moral attitude (mIAT), justice sensitivity (JSI), and subjective moral evaluations

**Fig. 3:**
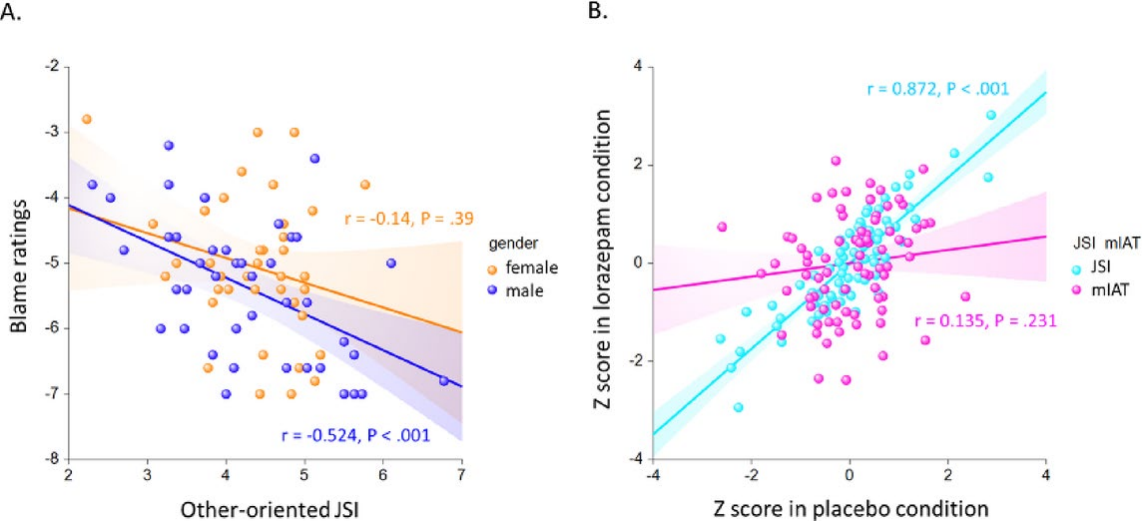
(**A**) The association between justice sensitivity and moral evaluation was modulated by the gender factor. While men showed a significant association between other-oriented JSI and blameworthy ratings for harming actions in lorazepam condition (*r* = -0.524, *P* < 0.001), women did not show this pattern of correlation (*r* = -0.14, *P* = 0.39). Fisher r-to-z transformation tests further corroborated the modulating role of gender on the justice sensitivity-moral evaluation relationship (△z = 1.91, *P* = 0.028, one-tailed). (**B**) The reliability of test-retest association between placebo and lorazepam condition was modulated by the implicit versus explicit measure of moral attitudes. While JSI showed a strong correlation between placebo and lorazepam condition (*r* = 0.872, *P* < 0.001), mIAT did not show this pattern (*r* = 0.135, *P* = 0.231). Fisher r-to-z transformation tests further corroborated the modulating role of implicit versus explicit moral attitudes on the placebo-lorazepam test-retest relationship (△z = 7.51, *P* < 0.001).

Parallel to previous findings^26,32^, participants who scored higher on the other-oriented Justice Sensitivity Inventory (JSI) assigned significantly more praise for helping (in the placebo condition: *r* = 0.321, *P* = 0.003; in the lorazepam condition: *r* = 0.355, *P* = 0.001) as well as more blame for harming (in the placebo condition: *r* = -0.355, *P* = 0.001; in the lorazepam condition: *r* = -0.312, *P* = 0.005), regardless of being in the placebo or lorazepam condition. However, neither blame nor praise ratings were associated with self-oriented JSI scores in either the placebo (blame: *r* = -0.141, *P* = 0.21; praise: *r* = 0.211, *P* = 0.059) or lorazepam condition (blame: *r* = -0.182, *P* = 0.104; praise: *r* = 0.187, *P* = 0.095). The relationship between justice sensitivity and moral evaluation was influenced by gender, with only men showing a significant association between other-oriented JSI and praise (in the placebo condition: *r* = 0.461, *P* = 0.002; in the lorazepam condition: *r* = 0.463, *P* = 0.002), and between other-oriented JSI and blame (in the placebo condition: *r* = -0.536, *P* < 0.001; in the lorazepam condition: *r* = -0.524, *P* < 0.001). In contrast, women did not demonstrate this pattern of association between justice sensitivity and moral evaluation (all *P* > 0.2). Fisher’s r-to-z transformation tests further confirmed the moderating effect of gender on the relationship between justice sensitivity and moral evaluation (Δz = 1.91, *P* = 0.028, one-tailed) (Fig. 3A). Implicit moral attitude (measured by the mIAT) did not significantly predict moral evaluation in any condition (i.e., placebo/lorazepam, male/female, and harming/helping) (all *P* > 0.2).

Regarding the effect of lorazepam on implicit moral attitude (mIAT) and justice sensitivity (JSI), while the total JSI score demonstrated a strong test-retest reliability between conditions (placebo and lorazepam) with a correlation of *r* = 0.872 (*P* < 0.001), the mIAT did not exhibit a similar pattern of correlation between the placebo and lorazepam conditions (*r* = 0.135, *P* = 0.231). Fisher’s r-to-z transformation tests further confirmed the distinct roles of implicit (mIAT) versus explicit (JSI) moral attitudes in influencing the test-retest relationship between the placebo and lorazepam conditions (Δz = 7.51, *P* < 0.001) (Fig. 3B). This finding aligns with previous ANOVA results, indicating that lorazepam had an overall effect in individuals with both low and high JSI scores (Fig. 2B), whereas its impact was only observed in participants with high baseline mIAT D scores. This differential effect of lorazepam on explicit and implicit moral attitudes was not influenced by the gender factor, as indicated by the correlation scores in female (JSI: *r* = 0.81, *P* < 0.001; mIAT: *r* = 0.098, *P* = 0.546) and male participants (JSI: *r* = 0.904, *P* < 0.001; mIAT: *r* = 0.202, *P* = 0.206).

## Discussion

This research, a double-blind, crossover, placebo-controlled study, explored the effects of lorazepam on moral judgment and attitudes, with a particular focus on the modulation of GABAergic potential. The research aimed to differentiate between self-interested and utilitarian behaviors using the Justice Sensitivity Inventory (JSI; self-oriented vs. other-oriented justice), and examined moral attitudes through both implicit and explicit measures. The key finding was that acute lorazepam administration did not significantly affect moral judgments overall, though changes in moral sensitivity appeared to vary with baseline levels, with greater reductions observed among individuals with higher baseline JSI and D scores. Specifically, participants with higher baseline values on the Implicit Association Test (mIAT) showed greater declines in D scores following lorazepam administration. A similar pattern was observed for both self- and other-oriented justice sensitivity, with participants exhibiting higher baseline JSI scores showing greater decreases as measured by the Justice Sensitivity Inventory. Furthermore, harmful scenarios were judged as more blameworthy and helping scenarios as more praiseworthy than neutral scenarios, though these judgments were unaffected by lorazepam administration.

One critical aspect of this study was the need to distinguish the effects of lorazepam on moral processing from its potential sedative side effects, given its known impact on psychomotor performance and reaction times (RTs)^37–39^. Therefore, RTs were used as an objective measure of sedation. If the acute administration of lorazepam did not specifically affect moral processing, it would be expected to evenly slow down RTs in both congruent and incongruent pairs of the mIAT, resulting in no effect on D scores. However, the study found that lorazepam’s effects varied depending on moral valence, suggesting that the observed changes were not solely due to sedation, and that lorazepam does specifically interact with moral processing mechanisms.

Although acute lorazepam administration did not significantly affect moral judgments in general, participants with higher baseline values on the mIAT exhibited greater declines in D scores following lorazepam administration. Higher D scores are associated with longer reaction times, indicating that individuals with lower D scores are quicker in their responses and possibly more attuned to their moral intuitions. Therefore, our findings appear to contradict earlier research, including the study by Martinez, et al.^31^, which suggested that individuals with lower D scores—often linked to those possessing the S allele in the 5-HTTLPR serotonin transporter polymorphism—relied more heavily on moral intuitions. Nevertheless, it is in line with studies showing that those with LL genotypes of the 5-HTT polymorphism—which tend to have less bioavailability of the neuromodulator serotonin in the synaptic cleft—reported less difficulty making harmful decisions, as they tend to incur in fewer negative emotions^40^. Research indicates that the upregulation of serotonin reinforces moral judgement by increasing aversion to harm^19^. Furthermore, while depletion of tryptophan—a serotonin precursor—elevates guilt in highly empathic individuals, it increases annoyance in those with high psychopathy traits. These findings suggest that serotonin fluctuation interacts with personality differences to produce diverse emotional responses^41^. The discrepancy may be further attributed to the different neurochemical systems involved—while 5-HTTLPR is related to the serotonergic system, lorazepam acts within the GABAergic system. Lorazepam might reduce anxiety-induced discomfort through the GABAergic system while preserving certain benefits like hypervigilance via the serotonergic system. However, previous studies have shown that serotonin transporters and GABAergic receptors are both linked to neuroticism^42^, with the former significantly affecting amygdala activity, a brain region where lorazepam is also known to exert significant inhibitory effects^43–45^. Despite this, the complex interplay between the GABAergic and serotonergic systems has not been definitively established^46,47^.

Notwithstanding, our findings concerning the interaction between mIAT D scores and lorazepam administration should be approached with caution. The test-retest reliability of the IAT ranges from *r* = 0.5 to 0.6^25^; however, in our study, the correlation of D scores between the placebo and lorazepam sessions was notably lower, at *r* = 0.135. Previous research indicates that implicit measures typically have lower stability over time compared to explicit measures across various domains (including self-concept, racial and political attitudes, among others), as they are more sensitive to contextual and situational influences, including social factors such as the presence of others or varying perspectives regarding agency^25–27^. Therefore, we contend that the low test-retest reliability observed in our mIAT findings may be indicative of the dynamic nature of implicit attitudes rather than a lack of reliability. This is particularly relevant following the administration of lorazepam, an anxiolytic drug observed to induce moral flexibility at the implicit level^3,12,28^. Additionally, lorazepam may affect different moral decision-making styles (e.g., deontological versus utilitarian), which are known to be context-dependent^3^; and potentially explaining its impact primarily on higher mIAT D scorers, which previous studies have associated with utilitarian moral decision-making^31^. Thus, these findings could be ascribed to certain selective effects of lorazepam. Nevertheless, and as previously stated, these findings should be interpreted with caution.

Regarding explicit justice sensitivity, lorazepam did not significantly alter moral sensitivity for either self- or other-oriented justice. However, these changes were significantly modulated by baseline levels of justice sensitivity. Participants with higher baseline scores tended to show greater declines in justice sensitivity following lorazepam administration. This trend toward reduced sensitivity to perceived injustice aligns with expectations, given lorazepam’s stress-, anxiety-, and sensitivity-reducing properties, as well as the explicit nature of the JSI, which captures consciously controlled moral cognition^34,48^. Although harm scenarios were consistently judged as more blameworthy and help scenarios as more praiseworthy than neutral ones—regardless of drug condition—the extent of sensitivity reduction may vary depending on individuals’ baseline levels. Additionally, the moral evaluations predicted by justice sensitivity—found to be greater in men than in women—may be partly attributed to socially desirable response tendencies, which can influence self-reported justice sensitivity and moral evaluations. Previous research has noted lower sensitivity to consequences among women^49,50^. While women consistently report more ethical responses than men, they are also more likely to respond in a socially desirable manner^51^. Thus, social desirability bias may significantly contribute to the observed relationship between gender and ethical decision-making^51^.

Our study found no interaction between Lorazepam administration, and D and JSI scores based on gender. Previous research has shown that in men and free-cycling women (i.e., not using oral contraceptives), LL homozygotes of the 5-HTTLPR serotonin transporter polymorphism exhibit reduced deontological tendencies compared to S allele carriers. In contrast, women on oral contraceptives with LL genotypes tend to make more deontological choices^40^. In this same line of research, intranasal administrations of oxytocin exhibited a differential effect based on gender. In male participants, intranasal oxytocin increased neural activity in disgust-related and self-processing regions, leading to more self-beneficial decisions in moral dilemmas, and contrary to oxytocin’s typical prosocial effects. In women, intranasal oxytocin enhanced self-processing activity but did not affect disgust responses. Women also showed a preference for other-directed rather than self-benefitting choices in moral dilemmas, aligning with prosocial behaviors^15^. It has been hypothesized that said selfish behavior in male participants could be due to oxytocin promoting in-group protection, where selfishness increases the survival chances of the in-group. This explanation would align with prior findings on oxytocin’s role in fostering in-group favoritism^52^. This is just to show that there is a complex interaction between gender, hormonal and endocrine systems, neuromodulator activity, and moral decision-making, such that it makes it challenging for our study to tease out these gender-based differences with the limited scope of our research.

Some limitations for this study should be acknowledged, particularly regarding the influence of individual differences and potential response biases in explicit moral judgments. Explicit measures can be affected by participants’ desire to please the experimenter or avoid endorsing socially undesirable behaviors. Cognitive load and the capacity to imagine different moral outcomes also play a role in shaping these judgments^53^. Therefore, post-session questionnaires assessing participants’ perceptions towards the experimenter could be useful for evaluating the influence of response expectancy in drug-placebo studies^54^. Secondly, we recognize that the test-retest reliability of the mIAT during lorazepam administration was low in our study. This finding aligns with the variability documented in previous research on implicit measures, which are known to be affected by contextual and situational factors^55^. However, we believe that further research is necessary to refine the methodology and evaluate its reliability under conditions specifically designed for this purpose. Last but not least, future research could benefit from including endophenotypic measures such as EEG or fMRI to better understand the mechanisms underlying lorazepam’s effects on moral cognition^56^.

In conclusion, this study reinforces previous findings that lorazepam, a GABA receptor agonist, may influence the underlying mechanisms of moral decision-making and moral cognition^3^, although without clear behavioral consequences. These findings offer a solid foundation for further investigations into the interactions between neuromodulatory drugs and moral cognition, paving the way for a deeper understanding of how pharmacological agents alongside their target neurotransmitters and neuropeptides impact ethical decision-making and social behavior.

## Electronic supplementary material

Below is the link to the electronic supplementary material.

Supplementary Material 1

## Data Availability

All data generated or analyzed during this study are available upon reasonable request to the corresponding author.
