# Supplementary material for: Acute lorazepam administration does not significantly affect moral attitudes or judgments
Source: Sci Rep. 2025 May 8;15:16108. doi: 10.1038/s41598-025-01109-9 (PMC12062430; doi:10.1038/s41598-025-01109-9)
Supplement: Supplementary file 1 — Supplementary Material 1 [file 41598_2025_1109_MOESM1_ESM.docx]

**Supplementary Table 1.**

**Justice Sensitivity Inventory: Sample Questions from each perspective.** This table illustrates examples for each of the four perspectives in the JSI –victim, observer, beneficiary and perpetrator, respectively. Respondents rate these items on a six-point scale ranging from 0 ('Not at all') to 5 ('Exactly'). The resultant scores reflect an individual’s sensitivity to moral norm violations and perceptions of injustice. Scores from the beneficiary, observer, and perpetrator perspectives are often aggregated to form a composite measure of other-oriented sensitivity.

| How do you react in unfair situations? | | |
| --- | --- | --- |
| People react quite differently in unfair situations. How about you? First, we will look at situations to the advantage of others and to your *own disadvantage*. | | |
| Not at all………….Exactly | | |
| 1. | It bothers me when others receive something that ought to be mine. | 0 1 2 3 4 5 |
| 2. | It makes me angry when others receive a reward that I have earned. | 0 1 2 3 4 5 |
| 3. | I cannot easily bear it when others profit unilaterally from me. | 0 1 2 3 4 5 |
| Now, we will look at situations in which you notice or learn that *someone else* is being treated unfairly, put at a disadvantage, or used. | | |
| Not at all………….Exactly | | |
| 1. | It bothers me when someone gets something they don’t deserve. | 0 1 2 3 4 5 |
| 2. | I am upset when someone does not get a reward he/she has earned. | 0 1 2 3 4 5 |
| 3. | I cannot easily bear it when someone unilaterally profits from others. | 0 1 2 3 4 5 |
| Now, we will look at situations that turn out to *your advantage* and to the disadvantage of others. | | |
| Not at all………….Exactly | | |
| 1. | It disturbs me when I receive what others ought to have. | 0 1 2 3 4 5 |
| 2. | I have a bad conscience when I receive a reward that someone else has earned. | 0 1 2 3 4 5 |
| 3. | I cannot easily bear it to unilaterally profit from others. | 0 1 2 3 4 5 |
| Finally, we will look at situations in which *you treat someone else unfairly*, discriminate against someone, or exploit someone. | | |
| Not at all………….Exactly | | |
| 1. | It gets me down when I take something from someone else that I don’t deserve. | 0 1 2 3 4 5 |
| 2. | I have a bad conscience when I deny someone the acknowledgment he or she deserves. | 0 1 2 3 4 5 |
| 3. | I cannot stand the feeling of exploiting someone. | 0 1 2 3 4 5 |

**Supplementary Figure 1.**

**Implicit Moral Attitude Test (mIAT): Sample Screens and Stimuli.** This figure illustrates example screens and stimuli from the mIAT. Panels (a), (b), (c), and (d) represent sample pairing tasks completed by participants. Short clips depicting moral or immoral actions, based on outcomes such as personal assistance or harm, along with words representing positive or negative evaluative attributes, are briefly presented at the center of the screen. Participants are instructed to quickly classify these stimuli into one of two category pairs displayed in the upper left and upper right corners of the screen, using the "z" or "/" keys on a computer keyboard. The relative speed to associate "moral + positive" and "immoral + negative" (panels a and b) compared to the opposite pairings, "moral + negative" and "immoral + positive" (panels c and d), determines the mIAT score. This score is interpreted as a measure of implicit moral attitudes.


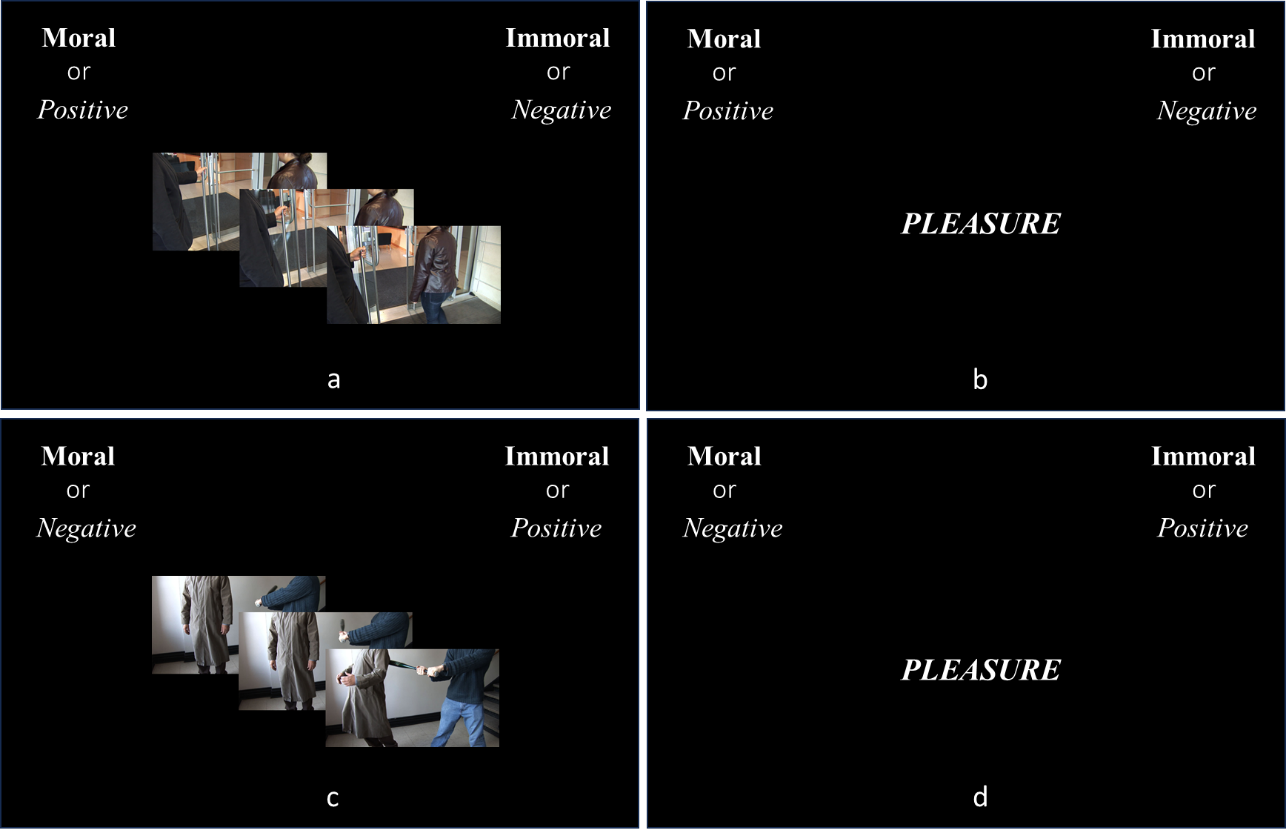


**Supplementary Figure 2.**


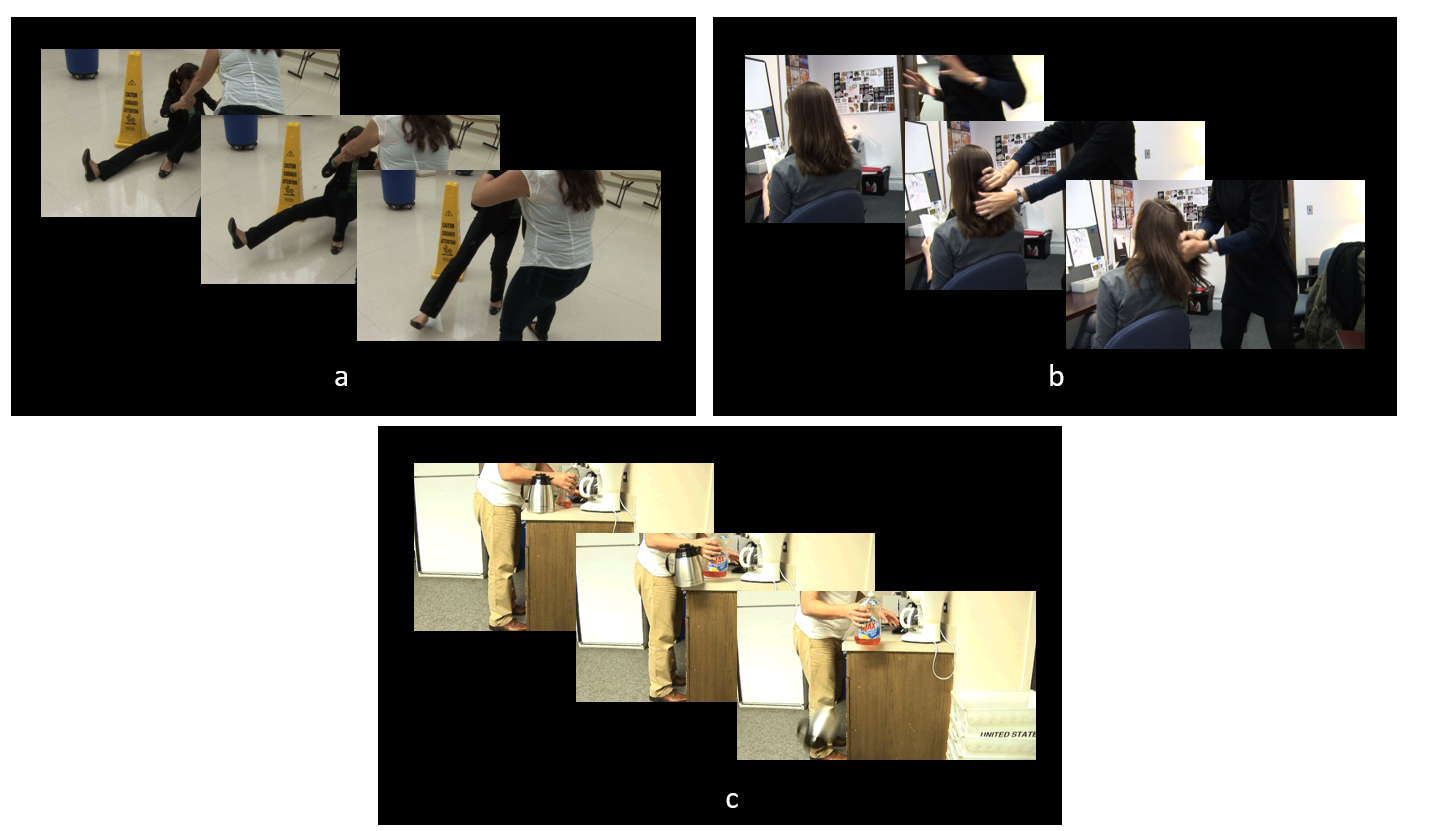
**Subjective Evaluations on Morally Laden Scenarios: Sample Screens and Stimuli.** This figure illustrates example screens and stimuli from the mIAT. Panels (a), (b), (c) represent samples for [1] a person alleviating another's physical pain (helping), [2] a person inflicting physical harm on another (harming), and [3] baseline stimuli depicting an action irrelevant to another person (neutral), respectively. In each animation trial, participants were asked to assess moral reasoning via a computer-based visual analogue scale. They rated each scenario on a Likert scale from “very much blameworthy (−7)”, through “neutral (0)”, to “very much praiseworthy (7).
